# A systematic review of decision analytic modeling techniques for the economic evaluation of dental caries interventions

**DOI:** 10.1371/journal.pone.0216921

**Published:** 2019-05-15

**Authors:** Zhi Qu, Shanshan Zhang, Christian Krauth, Xuenan Liu

**Affiliations:** 1 Institute for Epidemiology, Social Medicine and Health Systems Research, Hannover Medical School, Hannover, Germany; 2 Core Facility Quality Management and Health Technology Assessment for Transplantation, Hannover Medical School, Hannover, Germany; 3 Department of Preventive Dentistry, National Engineering Laboratory for Digital and Material Technology of Stomatology, Beijing Key Laboratory of Digital Stomatology, Peking University School and Hospital of Stomatology, Beijing, China; Western University, CANADA

## Abstract

**Objectives:**

Dental caries occur through a multifactorial process that may influence all tooth surfaces throughout an individual’s life. The application of decision analytical modeling (DAM) has gained an increasing level of attention in long-term outcome assessment and economic evaluation of interventions on caries in recent years. The objective of this study was to systematically review the application of DAM and assess their methodological quality in the context of dental caries.

**Methods:**

A systematic review of the literature published to 31^st^ December 2018 was conducted in Medline, EMBASE, NHSEED, and Web of Science electronic databases. The main information and model characteristics of studies was extracted with the methodological quality of included studies reviewed and assessed using the Philips’ checklist.

**Results:**

Twenty five studies from different settings were included. Modeling techniques mainly comprised main type of modeling including Markov models (n = 12), Markov/microsimulation mixed model (n = 7), systematic dynamic models (n = 3), microsimulation models (n = 2) and decision tree (n = 1). The mean number of criteria fulfilled was 25.1 out of 60 items, which varied between year of study and research groups. The percentage of criteria fulfilled for data dimension was lower than for the structure and consistency dimension. Critical issues were data quality, incorporation of utility values, and uncertainty analysis in modeling.

**Conclusion:**

The current review revealed that the methodological quality of DAM in dental caries economic evaluations is unsatisfied. Future modeling studies should adhere more closely to good practice guidelines, especially with respect to data quality evaluation, utility values incorporation, and uncertainty analysis in DAM based studies.

## Introduction

Dental caries is a multifactorial process that may influence all tooth surfaces throughout an individual’s life [1]. As one of the most prevalent chronic diseases [2–4], untreated dental caries affects 2.5 billion people worldwide with an all-age group combined prevalence of 34% [5]. Furthermore, caries are not only painful and give rise to discomfort but infection and systematic effects (i.e. sepsis) can occur when severe caries involve the pulp through dissemination of causative pathogens. Eventually, if untreated, permanent tooth decay and loss can result in disability. The World Health Organization (WHO) has estimated that caries accounted for almost 1.9 million disability adjusted life years (DALYs) globally in 2015, which is even more than the number caused by natural disasters [6]. Therefore, dental caries presents a considerable public health problem in both developed and developing countries.

Timely treatments have been shown to be cost-effective while prevention approaches such as fluoride toothpaste and water fluoridation are also of benefit. The caries intervention and prevention programs at both individual and population levels were launched many years ago, and the achievements of these programs on oral health are remarkable [7,8]. However, due to the long-term characteristics of caries that may exert influence throughout the life course, long-term outcome assessment and economic evaluation are necessary for decision-making when a number of interventions are available. Currently, health economic evaluations of all the interventions including non-pharmaceutical interventions require a long-term perspective in order to comprehensively assess their effects in comparison to real-life scenarios. Studies of long-term healthcare outcomes and cost-effectiveness exceeding a one-year follow-up period are seldom conducted when compared to clinical trials and short-term observational studies in this context.

Decision analytical modeling (DAM) is gaining increased attention in this field due to its capability in analyzing costs and consequences [9]. DAM comprises a series of approaches widely used in healthcare economics and outcome research, which can provide quantitative results to inform decision makers. Furthermore, DAM is a framework to permit synthesis of evidence from different sources and extends the time horizon beyond the follow-up period. DAM has been increasingly used in many healthcare systems and considered an indispensable approach for economic assessment of technology [10].

Mahl and colleagues [11] overviewed the use of Markov models in dental research in 2012, and several of the included studies focused on caries. However, the quality of the model and application of modeling techniques other than Markov models remains unknown even though a number of good practice DAM guidelines have been published [12–14]. High quality DAM is not only a scientific goal *per se* but should be consistent with the requirements of guidelines and support subsequent decision-making policies concerning the adoption of new technologies. Therefore, the objective of this study was to systematically review the DAM and assess the methodological quality of these in the context of dental caries. We aimed to offer insight into the utility of DAM while providing useful information, areas for quality improvement and recommendations for researchers. This would help to precipitate the outcomes from modeling more clearly and could be more attractive to decision-makers in this field.

## Methods

In accordance with the principles of the Cochrane Economics Methods Group (CCEMG) [15], we developed a review protocol and conducted a systematic review of the literature in the field of dental research.

### Literature search

The search strategy was developed by epidemiologists (ZQ and CK) and dental specialists (SZ and XL). A systematic review of the literature published to 31^st^ December 2018 was conducted in several electronic databases including Medline, EMBASE, National Health Service Economic Evaluation Database (NHS EED), and Web of Science (detailed search strategies are provided in S1 Table). The term “model” is based on the definition by the International Society For Pharmacoeconomics and Outcomes Research (ISPOR) Task Force on Good Research Practices—Modeling Studies: “an analytic methodology that accounts for events over time and across populations, that is based on data drawn from primary and/or secondary sources, and whose purpose is to estimate the effects of an intervention on valued health consequences and costs” [16].

### Eligibility criteria

This review included economic evaluation studies applying DAM techniques to assess dental caries interventions. Meanwhile, the included studies had to fulfill:

- Population: general population and selected sub-population having undergone caries interventions
- Intervention: healthcare intervention for prevention and treatment in the context of caries
- Comparator: no intervention or all alternative interventions
- Outcome: healthcare outcome using quality adjusted life years (QALYs) or quality adjusted tooth years (QATYs). Economic outcome including cost or benefit, incremental cost-effectiveness ratio (ICER)
- Study design: evaluation of long-term results and cost-effectiveness analysis

The language was restricted to English and no time restriction for when articles were published. We excluded descriptive studies and studies using models for illustration of the disease process or treatment options but without long-term benefits or cost measurements and non-economic studies in this field. Studies published as abstracts but without full information of the model were also excluded. We also performed manual examination of reference lists and citations of articles, and publications from other sources as a supplementary to the results.

### Review and data extraction

After combination and de-duplication of search results from databases, title and abstract screening were carried out on the basis of previously set inclusion and exclusion criteria. We then performed full text review to further exclude the ineligible studies. All included study information was systematically extracted into the pre-defined table. Literature review and data extraction were critically and independently performed by two authors (ZQ and SZ). Inconsistent judgments and data extraction results were collected and discussed with another author (XL) in order to reach consensus. When required information was missing or unclear, the authors were contacted to clarify the study details.

### Model assessment

As methods of DAM vary significantly and different approaches are needed for different problems requiring decisions, a complete standardization of models is difficult to infer. However, there are still well accepted guidelines worthy of referring to for good modeling practices. Philips and colleagues [17] have developed a framework for DAM quality assessment, which has already been widely applied in many health technology assessments for medical interventions. The Philips’ framework generally assesses the quality of DAM and its consistency with good practice guideline with respect to the three main dimensions: model structure, data, and consistency. Within each of the three dimensions, there are detailed criteria for checking whether the related information in the model was reported as fulfilled, not fulfilled, or not applicable. The detailed assessment of included models was initially performed by one author (ZQ) and reviewed by another author (SZ). We deliberately visualized the performance of the DAM in accordance with the good practice guideline, therefore we present the assessment results of included models with a bar chart on the basis of this checklist. The criteria for assessment are based on the example and explanation in Philips’ publication, and the ISPOR guideline for good practice [12].

## Results

### Search results

**Fig 1:**
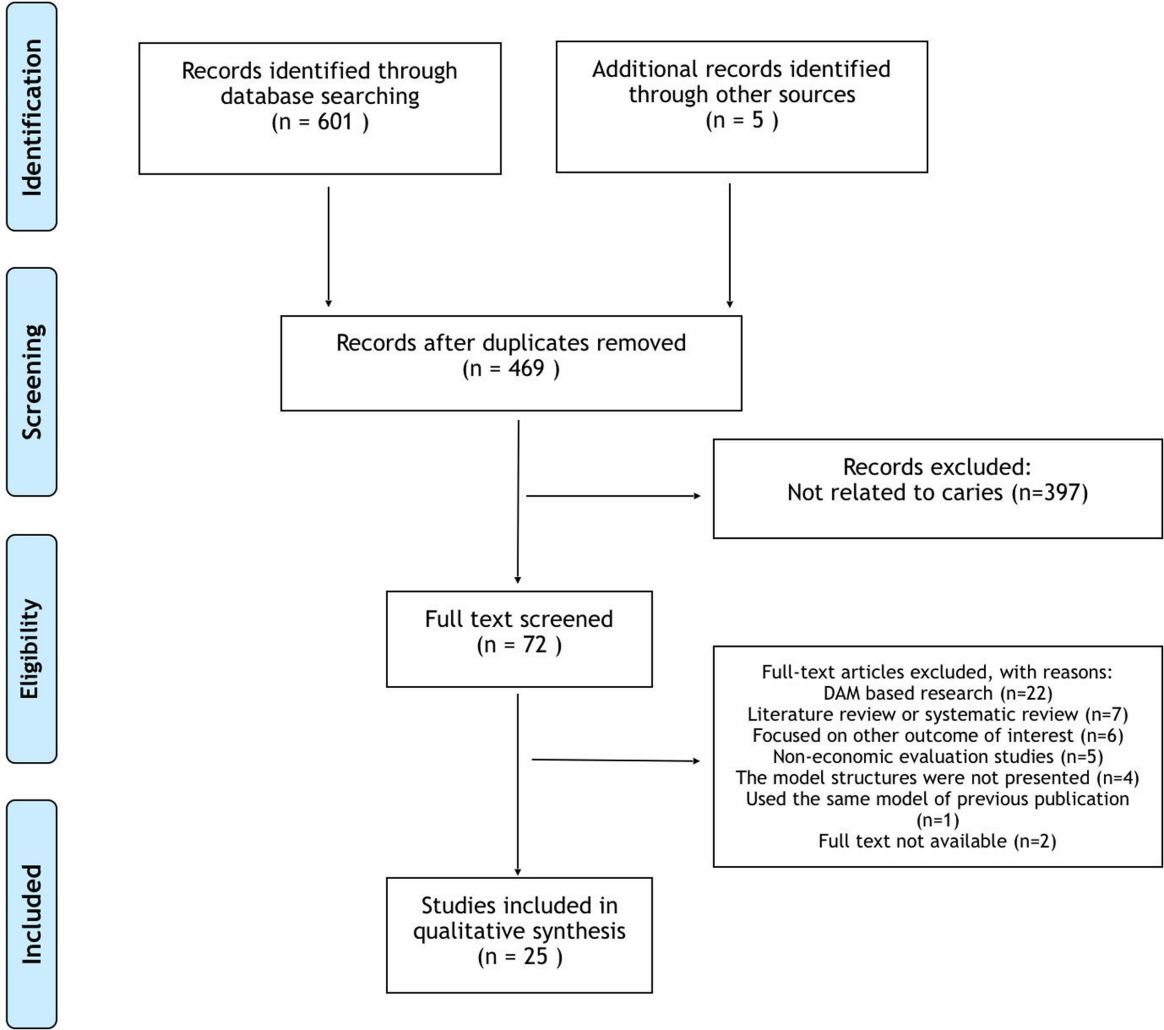
Flow chart of studies selection.

The database search identified 601 references and five additional ones were identified through hand search. After exclusion of 137 duplicates, 469 were further screened by title and abstract, 397 references were subsequently excluded as these were not related to dental caries. In further full text screening of remaining 72 references, studies were further excluded for the following reasons: non DAM based research (n = 22), literature review or systematic review (n = 7), focused on other outcome of interest rather than caries (n = 6), non-economic evaluation studies (n = 5), model structures were not presented (n = 4), used the same model of previous publication (n = 1). In addition, studies without available full text (n = 2) were excluded (S2 Table). After exclusion of 47 studies in total, 25 studies were finally included in the review [18–42]. The details of study selection are presented in Fig 1.

### Study characteristics

Most studies were conducted in Germany [22,27,29–31,34–37,40,42], followed by studies that were performed in the USA [18,20,25,28,32,41], in Australia [23,24,26,33,38], in the UK [19,39] and in Sweden [21]. The target populations were mainly school age children [18,21,22,28,34,35,37,41], pre-school aged children [20,25,33] and other specific populations [19,23,24,27–33,36,39,40,42]. Investigated strategies included preventive intervention [25,26,32,33,41,42], screening strategy [35,36], and invasive/non-invasive treatment [18–24,27–31,34,37,40]. General information of included studies is summarized in Table 1.

**Table 1:** General information of included studies.

Nr	Author	Year	Country	Target Population	Objective	Interventions and Comparators	Findings
**1**	Quinonez [18]	2005	USA	School-age children	Compare three strategies for managing the occlusal surfaces of first permanent molars	seal all, risk-based and seal none	Sealing children’s first permanent molars can improve outcomes and save money by delaying or avoiding invasive treatment and the destructive cycle of caries.
**2**	Brazzelli et al [19]	2006	UK	Typical patients	Assess the costeffectiveness of HealOzone for the treatment of caries	Current management and same strategy plus HealOzone	Insufficient to conclude that HealOzone is a cost-effective addition to the caries
**3**	Quinonez et al [20]	2006	USA	9–42 months children	Examine the cost-effectiveness of fluoride varnish application	application of universal fluoride varnish and no intervention	Fluoride varnish use in the medical setting is effective in reducing early childhood caries
**4**	Sköld et al [21]	2008	Sweden	School-age children	Analyze whether the fluoride varnish treatment and mouth-rinsing could result in cost containment in prevention of caries	Fluoride varnish treatment and fluoride mouth-rinsing	Prevention of approximal caries by fluoride varnish treatment may result in cost containment
**5**	Splieth et al [22]	2008	Germany	6-18y individual	Evaluate the economic consequences of caries prevention with fluorides	Caries prevention with and without fluorides	Use of fluorides in caries prevention is highly cost-effective
**6**	Warren et al [23]	2010	Australia	Australian population	Evaluates the long-term cost-effectiveness of the preventive approach	Caries Management System and standard dental care	Caries Management System is most cost-effective
**7**	Curtis et al [24]	2011	Australia	Caries patient	Assess the efficacy and cost-effectiveness of a non-invasive approach	Non-invasive Monitor Practice Programme and standard care	A joint preventive and non-invasive therapeutic approach appears to be cost-effective
**8**	Hirsch et al [25]	2012	USA	Preschool children	Determine interventions effects in reducing caries	Applying fluorides, limiting cariogenic bacterial transmission from mothers to children, using xylitol directly with children, clinical treatment, motivational interviewing and combinations of these	The systematic model can provide information to maximize the return on public health and clinical care investments
**9**	Pukallus et al [26]	2013	Australia	Low socioeconomic dental patients	Quantify the healthcare costs and the potential of cost saving	Telephone intervention and usual care	Telephone intervention generate considerable and immediate patient benefit and cost saving
**10**	Schwendicke et al[27]	2013	Germany	15-y individual	Analyze the long-term cost-effectiveness of incomplete and complete removal of deep caries	One- and two-step incomplete and complete excavations	One-step caries removal to be more cost-effective than both two-step incomplete and complete excavations of deep caries
**11**	Griffin et al [28]	2014	USA	Children in school	Estimate averted cavities using a minimal data set	Cavities with and without school-based sealant programs	Decision modeling provides an effective way to measure school-based sealant programs impact using a minimal data set
**12**	Schwendicke et al. [29]	2014	Germany	20-y individual	Compared the costs and effectiveness of alternative treatments of proximal caries lesions	Non-invasive, micro-invasive using resin infiltration, invasive using composite restoration	Non-and micro-invasive treatments have lower long-term costs than invasive therapy of proximal lesions
**13**	Schwendicke et al [30]	2014	Germany	18-y male individual	Compare the costs-effectiveness of different excavations	Selective, stepwise and complete excavation	Selective excavation seems most suitable to treat deep lesions
**14**	Schwendicke et al [31]	2014	Germany	20-y male individual	Assess the cost-effectiveness of treatment for pulps being exposed during caries removal	Direct pulp capping and root canal treatment	Direct pulp capping was more cost-effective in younger patients for occlusal exposure sites and root canal treatment was more effective in older patients or teeth with proximal exposures
**15**	Edelstein et al [32]	2015	USA	NY Medicaid population	Assess the potential for early childhood caries interventions to reduce cavity	Water fluoridation, fluoride varnish, fluoride toothpaste, medical screening and fluoride varnish application, bacterial transmission reduction, motivational interviewing, dental prevention visits, secondary prevention and combinations	The variety of population-level and individual-level interventions available to control ECC differ substantially
**16**	Koh et al [33]	2015	Australia	Children from age 6-months to 6-years	Evaluate the cost-effectiveness of a home-visit intervention and alternatives	Home-visit intervention, telephone based and no intervention	Home visits and telephone-based community interventions were highly cost-effective
**17**	Schwendicke et al [34]	2015	Germany	12-y individual	Assess the cost–effectiveness of radiographically and visually detection methods	Non-, micro-, or invasive treatments	Caries detection methods should be evaluated regarding the cost-effectiveness resulting from their use in different populations
**18**	Schwendicke et al [35]	2015	Germany	12-y individual	Assessed the cost-effectiveness of different detection methods for proximal secondary lesions	Combinations of visual-tactile, radiographic, or laser-fluorescence-based detection methods with 1 of 3 treatments initiated at different cutoffs	The suitability of detection methods differed significantly; the cost-effectiveness was greatly influenced by the treatment initiated after lesion detection.
**19**	Schwendicke et al [36]	2016	Germany	20-y individual	Assess the cost effectiveness of different detection method	Biannual tactile detection, radiographic detection every 2 years and biannual laser fluorescence detection	Current detection methods for secondary caries lesions should best be used in combination
**20**	Schwendicke et al [37]	2016	Germany	5-y children	Compare the cost-effectiveness of three strategies for treating primary molars	Conventional excavation and restoration, Hall Technique (caries sealing using a preformed crown) and pulpotomy	The Hall Technique was most cost-effective, whilst conventional treatment was least effective and more costly
**21**	Warren et al [38]	2016	Australia	Caries patient	Economic evaluation of the Caries Management System	Caries Management System and standard dental care	Caries Management System approach is effective and cost-effective compared with standard care
**22**	Hill et al [39]	2017	UK	Adult patients	Evaluate the cost-effectiveness of using mid-level providers instead of dentists	Usual care: dentist performed check-ups, direct restorations and endodontic treatment	Resources in public funded systems could be saved using mid-level providers in dental practices
**23**	Schwendicke et al [40]	2017	Germany	Population with different risk of caries	Assess the cost-effectiveness of root caries preventive treatments.	No treatment, fluoride rinses, chlorhexidine varnish and silver diamine fluoride varnish	Root caries preventive treatments (like silver diamine fluoride) are effective and might even be cost-saving in high risk populations.
**24**	Khouja et al [41]	2018	USA	Children over 9-year old	Compare the cost-effectiveness of pit and fissure sealants and fluoride varnishes in preventing dental caries	Pit and fissure sealants, fluoride varnishes and no intervention	Pit and fissure sealants should be the preferred method for the prevention of dental caries
**25**	Zimmer et al [42]	2018	Germany	12–74 years old German population	Analyze the lifetime monetary and health related effects of the consumption of sugar-free chewing gum	Sugar-free chewing gum consumption	Elevation of the consumption of sugar-free chewing gum would lead to a considerable benefit for cost saving and oral health for the statutory health insurance companies

The perspectives included mixed public-private payer [27,29–31,34–36,40], private dental practitioner [21,23,24,38,39], public payer [18,20,26,37,41] and societal perspectives [33]. The most common modeling techniques were the Markov model [18–20,24,26,28,31,33,38,39,41,42], followed by the Markov/microsimulation mixed model [27,29,30,34,35,37,40], systematic dynamic model [22,25,32], microsimulation model [23,36] and decision tree [21]. Time horizons ranged between 3.5 to 63.5 years when a life time horizon was not deployed. Cycle lengths ranged from one day to one year. Discount rates used were 3% [18,20,21,27–31,34–37,40–42], 3.5% [19], and 5% [22–24,26,38]. Fourteen of these discounting rates are for cost only [19,20,22,27,29–31,34–38,40,41], four for effect [21,28,32,42] and five for both cost and effect [18,23,24,26,33]. Baseline data were mainly derived from registry programs and surveys [19,22,23,28,33,42], cohort studies, [29,30,34,36] and the literature [18,21,27,31,35,36,38–41]. The treatment effect data were derived from the literature [18,21,29–32,34–36,38–41], randomized clinical trials (RCT) [23,24,37] and a single cohort study [33]. Most of the included studies had sensitivity analyses performed on key parameters and 17 studies run the model in more than one scenario to analyze the effect of variabilities [19,22–24,26,27,29–33,36–38,40–42]. Characteristics of the models in included studies are summarized in Table 2.

**Table 2:** Model characteristics.

Nr	Author	Perspective	Model Type	States	Time horizon	Cycle length	Discount rate	Source of data baseline	Source of data intervention	Sensitive analysis
**1**	Quinonez et al [18]	Payer	Markov model	Low risk sealed, low-risk not sealed, high risk sealed, high-risk not sealed, carious, restored.	10 years	1 month	3%	Literature	Literature and expert opinion	Univeriate sensitive analysis
**2**	Brazzelli et al [19]	NHS and Personal Social Services	Markov model	Progression of caries, reversal of caries, initial treatment repeated, tooth filled	5 years	1 year	3.5%	Data from NHS	NHS data and information from the manufacturer	One way sensitive analysis
**3**	Quinonez et al [20]	Medicaid payer	Markov model	No caries, caries, non-hospital treatment, hospital treatment	42 months	3 months	3%	Literature	Literature	Two way sensitive analysis
**4**	Sköld et al [21]	Dental care	Decision tree	No caries, enamel caries, dentin caries, filling	8 years	6 months	3%	Literature	Literature	One way sensitive analysis
**5**	Splieth et al [22]	NA	System dynamics model	Healthy, carious/one-surface filling, recurrent caries(two/three/four), surface filling, endodontics at four-surface filling, recurrent caries/crown, failure of crown/replaced with bridge	lifetime	1 day	5%	SHIP data	German National Health data	NA
**6**	Warren et al [23]	Private dental practitioner	Microsimulation	No disease, Enamel caries, Dentine caries, Filling, Repeat filling, Root canal, Crown, Extraction, Bridge, Implant and Death	lifetime	6 months	5%	AIHW data	Clinical trial	One-way sensitivity analyses
**7**	Curtis et al [24]	private dental practitioner	Markov model	No disease, enamel caries, dentine caries, filling, repeat, filling, root canal treatment, crown, bridge, extraction, implant and death	lifetime	6 months	5%	Dental claims data	Clinical trail	Univeriate sensitive analysis
**8**	Hirsch et al [25]	NA	System dynamics model	No caries activity, untreated caries, treated caries, symptomatic caries	10 years	NA	5%(inflation)	Colorado Child Health Survey	NHANES and MEPS data	NA
**9**	Pukallus et al [26]	Public health	Markov model	Early childhood caries, restoration, tooth restored within 6 months, restoration only, restoration without crown	5.5 years	6 months	5%	Logan-Beaudesert clinical database	Prevention programme data	Univeriate sensitive analysis
**10**	Schwendicke et al[27]	Mixed public-private payer	Markov model / Microsimulations	Remove of caries, root canal treatment, remove of tooth	63.5 years	6 months	3%	Literature	Private dental catalogue	Univariate sensitivity analyses
**11**	Griffin et al [28]	NA	Markov model	Sound sealed, sound unsealed, caries	9 years	1 year	3%	Data from Wisconsin Seal-A-Smile Program	Data from Wisconsin Seal-A-Smile Program	Two way sensitive analysis on tooth and program level
**12**	Schwendicke et al. [29]	Mixed public-private payer	Markov process model / Microsimulations	Radiographic extension into the enamel, outer third of the dentin, composite, complications, replacement with crown or gap	lifetime	6 months	3%	Cohort study	Non-systematic review	Univariate sensitivity analyses
**13**	Schwendicke et al [30]	Mixed public-private payer	Markov model / Microsimulations	Sound surface, shallow dentinal lesion, dental visit, treatment, untreated lesion deep dentinal lesion, shallow composite, refill, repair, second re-treatment, excavation, complication, re-treatment	lifetime	6 months	3%	Cohort study	Systematic review	Univariate sensitivity analyses
**14**	Schwendicke et al [31]	Mixed public-private payer	Markov model	Pulpal exposure, symptom, detection and treatment, development of symptom, complications and retreatment	lifetime	6 months	3%	Systematic review	Literature	Univariate sensitivity analyses
**15**	Edelstein et al [32]	NA	System dynamics modeling	NA	10 years	NA	NA	National Survey of Children’s Health	Literature and experts opinion	Sensitive analysis for MI group
**16**	Koh et al [33]	Societal	Markov model	Caries and healthy	5.5 years	6 months	5%	Data from ECC prevention programme	Original cohort study	Univariate and two way sensitive analysis
**17**	Schwendicke et al [34]	Mixed public-private payer	Markov model / Microsimulations	Sound or carious, extending into enamel or the outer or middle third of the dentine, false and true positive, treatment and no treatment, invasive, non- and micro-invasive detection	lifetime	6 months	3%	Literatures and Fourth German Oral Health Survey	Systematic review	Univariate and bivariate sensitivity analyses
**18**	Schwendicke et al [35]	Mixed public-private payer	Markov model / Microsimulations	Combinations of visual-tactile, radiographic, or laser-fluorescence–based detection methods with non-, micro-, and invasive treatment	lifetime	6 months	3%	Systematic review and KZBV report	Systematic review and meta-analysis	Univeriate and binary sensitive analysis
**19**	Schwendicke et al [36]	Mixed public-private payer	Microsimulations	False positive, true positive, treatment, no treatment, re-store with composite/crown, lesion development, pulp exposure, progression to pulp disease, direct capping, root canal treatment, restore, crown, extraction	lifetime	6 months	3%	Systematic review and cohort data reporting	Systematic review	Univeriate and binary sensitive analysis
**20**	Schwendicke et al [37]	Public payer	Markov model / Microsimulations	Restorative minor complication, restorative major or any pulp complication, replacement or repair, removal	7 years	6 months	3%	Primary trials	Primary trials and meta-analysis	One way microsimulation sensitive analysis
**21**	Warren et al [38]	Private dental practitioner	Markov model	No disease, enamel caries, dentine caries, filling, repeat, filling, root canal treatment, crown, bridge, extraction, implant and death	lifetime	6 months	5%	Data from the AIHW	Original RCT	Univeriate sensitive analysis
**22**	Hill et al [39]	Practice owners	Markov model	Health and caries	5 years	6 months	NA	Literature and study report in NHS	Literature and study report in NHS	One and Two-way sensitivity analyses
**23**	Schwendicke et al [40]	Mixed public-private payer	Markov model / Microsimulations	Tooth without caries, treated, after treated fail or success	10 years	1 year	3%	Systematic review and meta-analysis	Systematic review and meta-analysis	Univeriate sensitive analysis
**24**	Khouja et al [41]	Payer	Markov model	Sound tooth and carious tooth	9 years	1 year	3%	Literature review and meta-analysis	Literature review and meta-analysis	One and multiple way sensitivity analyses
**25**	Zimmer et al [42]	German statutory health insurance system	Markov model	No caries, filled teeth, crown, Bridge/Prosthesis/Implant	62 years	1 year	3%	Official statistical databases and literature review	Official statistical databases	NA

AIHW: Australian Institute of Health and Welfare, MEPS: Medical Panel Expenditure Survey, NHANES: National Health and Nutrition Examination Survey, NHS: National Health Service, SHIP: Study of Health in Pomerania, KZBV: National Association of Statutory Health Insurance Dentists (Kassenärztlicher Bundesvereinigung),

### Quality assessment

**Fig 2:**
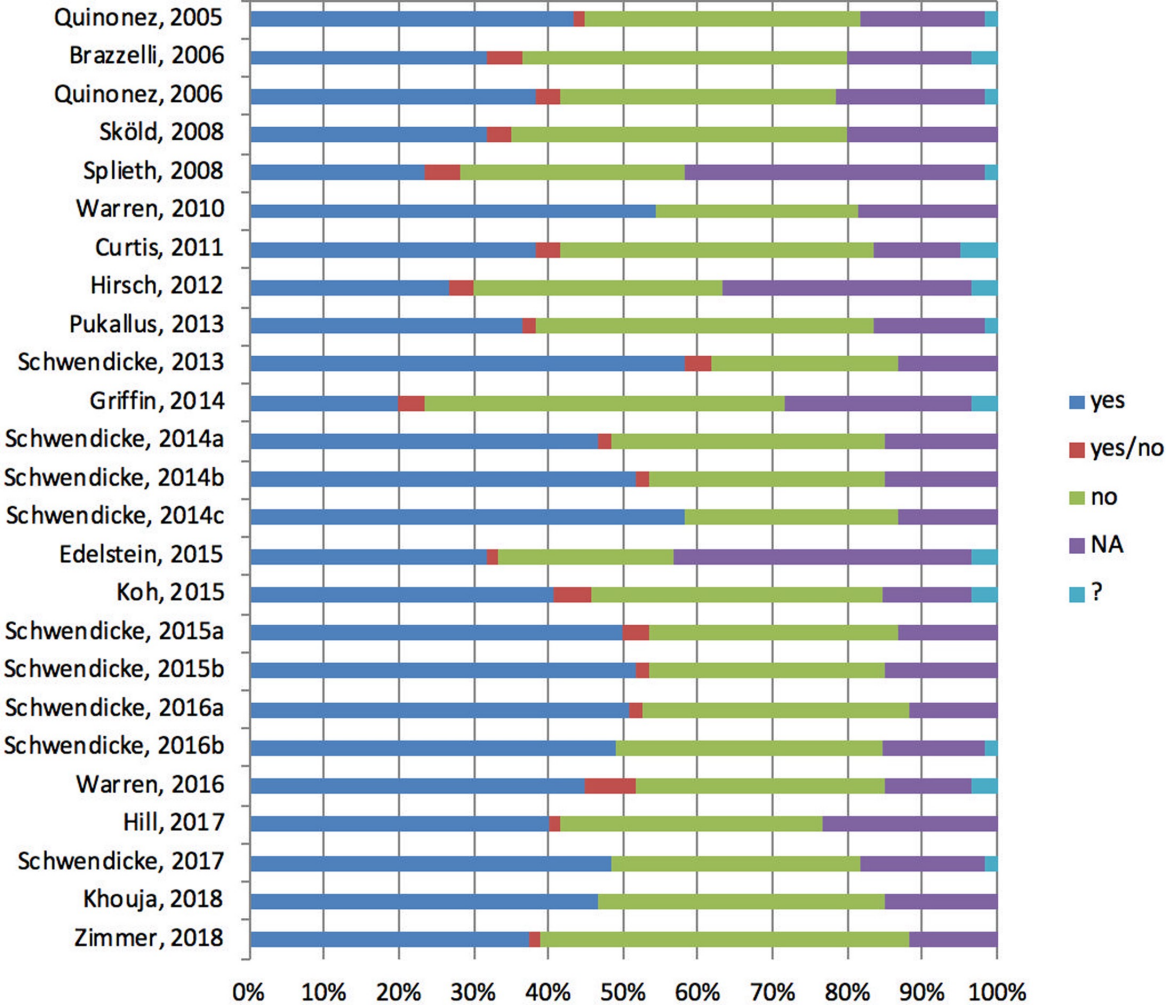
Methodological evaluation of included studies. Methodological quality of included studies according to the criteria of Philips et al. (Philips, 2006). A ‘yes’ answer represents if a question was fulfilled, ‘no’ represents that is not fulfilled. ‘yes/no’ represents if a question was consisted of two sub-questions, one was fulfilled but one was not. ‘NA’ was assigned when the answer is not applicable. ‘?’ was assigned when the answer was not clear for assessment.

The results of the methodological quality assessment of included studies are shown in Fig 2. The mean number of fulfilled criteria were 25.1 out of 60 (median 26, range 12–35). Altogether, the percentage of criteria fulfilled varied between year of study and research groups. Furthermore, the percentage of criteria fulfilled for data section was lower than the structure and consistency section. Critical issues were data quality, incorporation of utility values, and uncertainty analysis in models. More detailed information for quality assessment is available in the supporting information (S3 Table).

### Structure

All included studies clearly stated the decision aims and had a consistent objective. However, none of the studies specified the main decision-maker. Most studies clearly stated the perspective except five [19,22,25,28,32]. Therefore, it was questionable whether the input of the model was consistent with the scope of the aims of these studies. Nine of the included studies [18,19,22,23,25,28,32,37,41] introduced evidence regarding model structure but none of these considered other theories of model structure. All included studies specified the source of data except for one [19], and only in one study was it not clear what the rationale was for the type of model [22]. Over half of the included studies used a lifetime horizon [22–24,27,29–31,34–36,38] or “almost lifetime” horizon [27,42] but none of the remaining studies justified the use of a shorter time horizon. In five studies [24,28,32,33,38] disease states or pathway were not explicitly stated.

### Data

Quality assessment of data was not performed in any of the included studies. Over half of the included studies [18,21,23,24,27,29–31,35,37–39,41] analyzed the data using a justifiable epidemiological technique in the pre-model analysis. Calculation of transition probabilities were not justified in six studies [18,20–22,25,38]. Only one study [33] mentioned the deviation for utility index and justified the utilization. None of the studies addressed all types of uncertainties and only one of them [38] justified the omission of a particular form. One study [38] addressed the heterogeneity by running the model separately for different populations.

### Consistency

Only two studies [22,28] were tested for mathematical logic before use, and three studies clearly stated that their models [22,29,32] were calibrated against independent data. No counter-intuitive results were reported thus no further explanation and justification was given. In addition, over half of the included studies compared their results to the previous DAMs used in other studies.

## Discussion

Our study systematically reviewed decision analytical models in the published literature for economic evaluations in the field of caries. This review brings all applied models together and thus provides helpful information regarding the current situation for dental healthcare givers, while also identifying aspects that could be improved in future modeling studies. To date, this is the first systematic review that depicts the characteristics of decision analytical models used in this field and quantitatively assessed the methodological quality with the widely used Philips’ checklist. The methodological quality of the models is comparable with the findings from peers’ studies in other areas as acute coronary syndrome [43] and lower extremity artery disease [44] but still unsatisfying.

The modeling approach types in the included studies were mainly Markov models or Markov-based microsimulation models. The characteristics of Markov models could provide a framework to represent sequences of events and effects (such as the demineralization–remineralization dynamic processes in teeth surface) of treatment over time. Compared with the widely used decision-tree model in the context of other oral diseases, patients with caries may experience the disease and intervention process more frequently, thus state transition models such as Markov models could represent the condition more realistically. Besides, use of microsimulation models could provide a way of keeping track of the natural history of certain patients, which more accurately models the interaction within populations and their environment over time. Such models are more suitable when community intervention is performed e.g., water or salt fluoridation. However, there is no general classification that connects decision problems and modeling type. Different caries intervention evaluations require different modeling approaches. Markov models have also been used in clinical and service evaluation of dental diseases, such as implantology for decision-making [45], prosthodontic dentistry for treatment cycles [46], and dental health services [47]. Selection of the modeling method should be based on the natural history of the disease and characteristics of intervention alternatives, model appropriateness, dimensions, ease and speed of model development, and data sources [48].

As mentioned above, the quality and availability of data is another key element in modeling studies. However, assessment of the data quality was not conducted in any of the included studies within this systematic review. Choosing the right input parameters and assuring its accuracy is as important as conceptualizing an appropriate model structure when conducting analyses [49]. Data sources of included studies are mainly local retrospective studies and literature reviews. Although results derived from RCTs are more reliable in quantifying the effect of interventions, observational evidence (e.g., disease registries and administrative claim data) may be more useful in estimating the natural progression of the disease, resource use/cost, and utility data [50]. Moreover, observational data can be used to mitigate against the shortage of RCT data as it might miss interested events or combine the endpoints as outcomes [43]. Published literature is an important aggregate data source for modeling, which was adapted by most of the included studies in this review. In the current review, two thirds of all included studies performed systematic literature reviews for more accurate input parameters, which is in accordance with the recommendations in the good practice guideline. Systematic reviews and meta-analysis are useful approaches to solving disagreements in the literature, at least for important effectiveness parameters or the main clinical effects of interests. However, for many researchers, it is not feasible to perform a systematic review because of the restriction of time and other resources, thus an explanation for choice of data source should be made to assure the reliability and validity of the model [43]. Expert opinion was adapted in one study, which was used as an additional data source. Although expert opinion is the least preferred data source because of the subjectivity, it is still worth considering when clinical treatment information is not available. Under the decision analytical model framework, all available sources of information should be integrated to achieve the most appropriate model.

The perspective determines the input parameters and measurement of costs in DAM studies. The societal perspective which aims to include all type of costs is generally considered as the most appropriate point of view for decision makers [9]. However, it is worth noting that only one study in current research stated a societal perspective clearly and none of the included studies define the decision makers. Although there is no optimal choice among these different and contradicted perspectives, clarifying the decision maker before modeling analysis and choosing corresponding perspective is highly recommended on the basis of current findings.

The employment of QALYs as the measurement of outcomes allows the comparison of different interventions with clinical outcomes cross the DAM based studies from different area. Although the oral symptoms such as toothache and consequent poor nutritional intake has significantly detrimental influences on the quality of life of patients [1], some researchers believe it was not possible to measure health benefits in terms of QALYs for dental caries treatment [19], thus the cost-effectiveness measurement of utility in dentistry, which was analogous to QALYs was to calculate the year of single tooth (i.e. quality adjusted tooth years, QATYs) as proposed by Birch and colleagues [51]. Despite using QATYs may limit the comparability of results, we recommend the use of QATYs as outcome measures for DAM or other economic evaluations in the field of dentistry, especially if the decayed, missing, and filled teeth or surface (DMFT/S) is used as the outcome, in order to achieve more accurate and practical outcome measurement. To our best knowledge, there are few DAM based studies using QATYs for outcome measurement, therefore further studies should also focus on improvement of the oral health-related quality of life measurement, while also considering the school or workplace absence, and increased risk of hospitalization.

Uncertainty is the fabric of modeling and statistics [52]. Although more than one guideline recommends uncertainty analysis in methodological, structural population heterogeneity and parameter levels, most of the included studies performed sensitivity analyses only for important parameters, while none of the studies addressed other aspects of uncertainty. As accounting for uncertainty has become a standard part of decision analytic modeling, we recommend that more comprehensive uncertainty analysis should be considered in further modeling practice, in order to improve the methodological quality and accuracy. A step-by-step practical guide developed by Bilcke and colleagues [53] could be a useful reference for assessment.

In addition to the practical guide, communication and collaboration between researchers who are interested to take first steps in DAM studies and experienced researchers is crucial. One research group performed almost one third of included studies and dominated the research work in this area which demonstrates that there is a huge lack of DAM studies in this area. We hope the current research provide a comprehensive overview of and encourage more researcher to participate into the development of DAM in this area.

### Limitations

It is noteworthy that a major limitation of the current research is that our review on model structure and evaluation of model quality is based on published literature and appendices, while technical reports of the studies included were not publicly available. Although the guideline for model construction and good practice were published decades ago, due to the space constraint of academic journals, authors cannot fully describe the details and extensive consideration of their models, and how these fulfill the guideline. Our communication with authors did not make all items in the checklist clear; this was limited by the approach and style of communication itself, which may lead to an overestimation of the methodological quality. This may affect the results of the model evaluation, thus we recommend that the technical reports of modeling studies could be published as an appendix or be open to the researchers who are interested in secondary study, which would permit transparency of communication into practice. Another limitation is the publication bias. Though manual searches were conducted to further enhance the electronic database search, the included publications in the current review were published in peer-reviewed journals only, and some relevant reports in the grey literature may have been missed. It could be eliminated when all economic studies were registered before the start as clinical trial. Currently, publication bias cannot be ruled out.

## Conclusion

Based on the current systematic review of decision analytic models for prevention and treatment of caries, we conclude that in most studies, Markov models were applied to simulate the progress of disease and effectiveness of interventions. Although the qualities of models are acceptable according to the Philips checklist, the percentage of criteria that were fulfilled varied between studies.

We recommend that decision analytical modeling in the context of economic evaluation of interventions for caries considers an assessment of the data quality and appropriately incorporate utility values. Furthermore, special emphasis should be given to uncertainty analysis, which is important for the transparency and communication of modeling. Practical guidelines are worth referring to when performing uncertainty analysis. Appropriate model structure and systematic evaluation of data together with adherence to good practice guidelines will inform the healthcare provider in allocating resources when novel interventions emerge in the future.

## Supporting information

S1 Table Search strategy. (DOCX)

S2 Table Information on excluded studies. (XLSX)

S3 Table Methodological evaluation of included studies. (XLSX)

S4 Table PRISMA checklist. (DOC)
