# Supplementary material for: A systematic review of decision analytic modeling techniques for the economic evaluation of dental caries interventions
Source: PLoS One. 2019 May 15;14(5):e0216921. doi: 10.1371/journal.pone.0216921 (PMC6519822; doi:10.1371/journal.pone.0216921)
Supplement: S1 Table — (DOCX) [file pone.0216921.s001.docx]

S1 Table. Searching strategy

| **No.** | **Term** |
| --- | --- |
| 1 | dentistry[MeSH Terms] |
| 2 | oral health[MeSH Terms] |
| 3 | dental[MeSH Terms] |
| 4 | tooth[MeSH Terms] |
| 5 | or 1-4 |
| 6 | decision analytic model* |
| 7 | decision model* |
| 8 | markov model* |
| 9 | markov cohort model* |
| 10 | decision tree* |
| 11 | microsimulation* |
| 12 | simulation model* |
| 13 | state transition model* |
| 14 | discrete event simulation* |
| 15 | dynamic model* |
| 16 | or 6-15 |
| 17 | cost benefit analysis/ |
| 18 | cost effect* |
| 19 | cost utilit* |
| 20 | cost benefi* |
| 21 | economic analy* |
| 22 | economic evaluat* |
| 23 | or 17-22 |
| 24 | QALY |
| 25 | quality adjusted life |
| 26 | quality adjusted days |
| 27 | quality adjusted month |
| 28 | quality adjusted survival |
| 29  30 | quality adjusted year$.  QATY |
| 31 | quality adjusted tooth year |
| 32 | or 24-31 |
| 33 | ICER |
| 34 | incremental ratio |
| 35 | or 33-34 |
| 36 | 23 or 30 or 35 |
| 37 | 5 and 16 and 36 |
| 38 | Search English[Language] |
| 39 | Search human[MeSH Terms] |
| 40 | 37 and 38 and 39 |
